# Adolescent Wound-Care Self-Efficacy and Practices After Voluntary Medical Male Circumcision—A Multicountry Assessment

**DOI:** 10.1093/cid/cix953

**Published:** 2018-04-03

**Authors:** Webster Mavhu, Karin Hatzold, Kim H Dam, Michelle R Kaufman, Eshan U Patel, Lynn M Van Lith, Catherine Kahabuka, Arik V Marcell, Lusanda Mahlasela, Emmanuel Njeuhmeli, Kim Seifert Ahanda, Getrude Ncube, Gissenge Lija, Collen Bonnecwe, Aaron A R Tobian

**Affiliations:** 1 Centre for Sexual Health & HIV/AIDS Research, Harare, Zimbabwe; 2 Population Services International, Harare, Zimbabwe; 3 Johns Hopkins Center for Communication Programs, Baltimore, MD; 4 Johns Hopkins Bloomberg School of Public Health, Baltimore, MD; 5 Department of Pathology, Johns Hopkins School of Medicine, Baltimore, MD; 6 CSK Research Solutions, Ltd., Dar es Salaam, Tanzania; 7 Department of Pediatrics, Johns Hopkins School of Medicine, Baltimore, MD; 8 Centre for Communication Impact, Pretoria, South Africa; 9 Office of HIV/AIDS, Global Health Bureau, United States Agency for International Development, Washington, DC; 10 Ministry of Health and Child Care, Harare, Zimbabwe; 11 Ministry of Health, Community Development, Gender, Elderly and Children, Dar es Salaam, Tanzania; 12 National Department of Health, Pretoria, South Africa

**Keywords:** voluntary medical male circumcision, HIV, adolescents, wound-care, sub-Saharan Africa

## Abstract

**Background:**

Adolescent boys (aged 10–19 years) constitute the majority of voluntary medical male circumcision (VMMC) clients in sub-Saharan Africa. They are at higher risk of postoperative infections compared to adults. We explored adolescents’ wound-care knowledge, self-efficacy, and practices after VMMC to inform strategies for reducing the risks of infectious complications postoperatively.

**Methods:**

Quantitative and qualitative data were collected in South Africa, Tanzania, and Zimbabwe between June 2015 to September 2016. A postprocedure survey was conducted approximately 7–10 days after VMMC among male adolescents (n = 1293) who had completed a preprocedure survey; the postprocedure survey assessed knowledge of proper wound care and wound-care self-efficacy. We also conducted in-depth interviews (n = 92) with male adolescents 6–10 weeks after the VMMC procedure to further explore comprehension of providers’ wound-care instructions as well as wound-care practices, and we held 24 focus group discussions with randomly selected parents/guardians of the adolescents.

**Results:**

Adolescent VMMC clients face multiple challenges with postcircumcision wound care owing to factors such as forgetting, misinterpreting, and disregarding provider instructions. Although younger adolescents stated that parental intervention helped them overcome potential hindrances to wound care, parents and guardians lacked crucial information on wound care because most had not attended counseling sessions. Some older adolescents reported ignoring symptoms of infection and not returning to the clinic for review when an adverse event had occurred.

**Conclusions:**

Increased involvement of parents/guardians in wound-care counseling for younger adolescents and in wound-care supervision, alongside the development of age-appropriate materials on wound care, are needed to minimize postoperative complications after VMMC.

Adolescents (aged 10–19 years) constitute the majority of voluntary medical male circumcision (VMMC) clients in sub-Saharan Africa [1–3]. VMMC priority countries aim to offer VMMC and circumcise 90% of boys and men aged 10–29 years by 2021 in line with Joint United Nations Programme on HIV and AIDS (UNAIDS) human immunodeficiency virus (HIV) prevention Fast Track goals [4]. As VMMC scale-up advances, provision of high-quality services is essential to maintain client safety, increase acceptability, and ensure program impact [5].

The proportion of clients experiencing adverse events (AEs) remains a widely used indicator of program quality [5]. Because proper wound care minimizes VMMC-related postoperative AEs, the World Health Organization (WHO) and the US President’s Emergency Plan for AIDS Relief (PEPFAR) outline key postoperative messages that providers should communicate to clients to ensure proper wound care [6–8]. These include the need for clients to use clean water to wash wounds, desist from applying home remedies and traditional medicines, abstain from sexual intercourse and masturbation for 6 weeks, and contact a health provider and/or visit a clinic in case of complications [6–8].

Although VMMC-related AEs have remained low, WHO has reported an increase in infection-related AEs, including severe ones in sub-Saharan Africa [9]. These include cases of postoperative wound infection with tetanus after both surgical (conventional) and device-enabled VMMC. Inappropriate wound-care practices, including application of substances, such as animal dung poultices or herbal remedies that could contain tetanus bacterial spores, contribute to infection [9]. Postoperative wound infection remains the most common postprocedure AE, particularly among adolescents [5, 10–12]. For example, in Zimbabwe, compared with clients aged ≥20 years, those aged 10–14 or 15–19 years were 3.1 and 1.8 times more likely to experience an infection, respectively [5].

WHO has emphasized the need to mitigate post-VMMC infection risk, especially through ensuring proper client wound management [9]. The current study explored adolescents’ wound-care knowledge, self-efficacy, and practices after VMMC to inform strategies for reducing the risks of infection complications postoperatively.

## METHODS

### Study Setting and Design

Quantitative and qualitative data were collected in South Africa, Tanzania, and Zimbabwe between June 2015 and September 2016. We undertook a quantitative postprocedure survey approximately 7–10 days after VMMC at 14 health facilities (South Africa, n = 4; Tanzania, n = 4; Zimbabwe, n = 6) with adolescent VMMC clients who had completed a preprocedure survey. Study procedures are described in detail elsewhere [13, 14]. Analysis of quantitative data was limited to participants who completed the follow-up survey, at which time we measured adolescents’ wound-care self-efficacy and knowledge of proper wound-care practices.

Qualitative in-depth interviews (IDIs), conducted with male adolescents (aged 10–19 years) 6–10 weeks after the VMMC procedure, explored comprehension of providers’ wound-care instructions and wound-care practices. Focus group discussions (FGDs), separated by gender, included parents/guardians from communities surrounding the 14 sites whose adolescents had agreed to be circumcised or had been recently circumcised. Structured interviews, IDIs, and FGDs were conducted in Sesotho, isiZulu, or isiSwati in South Africa; KiSwahili in Tanzania; and Shona or Ndebele in Zimbabwe. IDIs and FGDs were audio recorded, transcribed, and translated into English for coding and analysis.

### Data Analysis

Descriptive statistics were used to examine characteristics of the study population and responses obtained during the structured quantitative surveys. Qualitative data were analyzed by 2 coders using a 2-step process. All transcripts were first coded independently with predetermined areas of inquiry, and coders developed appropriate categories and subcategories as needed. All transcripts were double coded, with the 2 coders discussing any coding discrepancies until a consensus was met, as described elsewhere [15]. Quantitative and qualitative analyses were performed using STATA SE (version 14; StataCorp) and ATLAS.ti (version 7) software, respectively.

### Ethical Approval

Ethical approval was obtained from the Human Sciences Research Council in South Africa, the Tanzania National Institute for Medical Research, the Medical Research Council of Zimbabwe, and the Johns Hopkins Bloomberg School of Public Health Institutional Review Board before data collection. All participants provided informed consent or assent (with parental consent).

## RESULTS

### Study Population

A total of 1293 male adolescents in South Africa (n = 299), Tanzania (n = 498), and Zimbabwe (n = 496) took part in structured interviews approximately 7–10 days after the VMMC procedure. Ninety-two additional adolescents (South Africa, n = 36; Tanzania, n = 36; Zimbabwe, n = 20) were selected for IDIs 6–10 weeks after the VMMC procedure. In addition, 192 female and male parents/guardians participated in 24 FGDs (8 FGDs per country with 6–12 participants per FGD) in South Africa (n = 59), Tanzania (n = 60), and Zimbabwe (n = 73). See Table 1 for sociodemographic characteristics of the adolescents and their parents/guardians.

**Table 1.** Participant Characteristics by Country

Characteristic	Participants, No. (%)^a^			
	All Countries	South Africa	Tanzania	Zimbabwe
**Structured quantitative survey (adolescents**)	**n = 1293**	**n = 299**	**n = 498**	**n = 496**
Age, mean (SD), y	13.5 (3.1)	13.8 (3.0)	12.0 (2.6)	14.7 (3.0)
Age group, y				
10–14	836 (64.7)	187 (62.5)	413 (82.9)	236 (47.6)
15–19	457 (35.3)	112 (37.5)	85 (17.1)	260 (52.4)
Setting				
Urban	696 (53.8)	107 (35.8)	233 (46.8)	356 (71.8)
Periurban	192 (14.9)	50 (16.7)	142 (28.5)	0 (0.0)
Rural	405 (31.3)	142 (47.5)	123 (24.7)	140 (28.2)
**Qualitative IDIs (adolescents**)	**n = 92**	**n = 36**	**n = 36**	**n = 20**
Age, mean (SD), y	14.5 (2.9)	13.4 (2.3)	15.1 (3.4)	15.5 (2.3)
Age group, y				
10–14	49 (53.3)	28 (77.8)	15 (41.7)	6 (30.0)
15–19	43 (46.7)	8 (22.2)	21 (58.3)	14 (70.0)
Setting				
Urban	55 (59.8)	9 (25.0)	31 (86.1)	15 (75.0)
Periurban	14 (15.2)	9 (25.0)	5 (13.9)	0 (0.0)
Rural	23 (25.0)	18 (50.0)	0 (0.0)	5 (25.0)
**Qualitative FGDs (parents/guardians**)	**n = 192**	**n = 59**	**n = 60**	**n = 73**
Age, mean (SD), y	39.9 (11.5)	40.5 (10.1)	36.1 (10.1)	42.5 (12.8)
Gender				
Female	97 (50.5)	30 (50.8)	31 (51.7)	36 (49.3)
Male	95 (49.5)	29 (49.2)	29 (48.3)	37 (50.7)
Setting				
Urban	89 (46.4)	18 (30.5)	16 (26.7)	55 (75.3)
Periurban	43 (22.4)	13 (22.0)	30 (50.0)	0 (0.0)
Rural	60 (31.3)	28 (47.5)	14 (23.3)	18 (24.7)
Completed primary education				
No	31 (16.2)	18 (30.5)	5 (8.3)	8 (11.0)
Yes	161 (83.9)	41 (69.5)	55 (91.7)	65 (89.0)

Abbreviations: FGDs, focus group discussions; IDIs, in-depth interviews; SD, standard deviation.

^a^Data represent No. (%) of participants unless otherwise specified.

### Knowledge, Recall, and Comprehension of Proper Wound-Care Instructions

Table 2 shows adolescents’ knowledge of proper wound-healing practices 7–10 days after the VMMC procedure. Overall, knowledge was suboptimal, but some adolescents did repeat key postoperative messages outlined in WHO and PEPFAR guidelines [6–8], suggesting that providers had mentioned these during counseling. Frequently cited instructions included the need to abstain from sex (44.6%) and masturbation (24.7%), avoid physical activities (19.5%), refrain from touching the penis (18.8%), change the bandage (17.3%), and go back to the clinic for a checkup (13.6%). The need to desist from applying home/traditional remedies was mentioned by only 5.0%.

**Table 2.** Knowledge of Proper Wound-Care Practices Among Adolescents Surveyed Approximately 7–10 Days After VMMC Procedure

Practice to Ensure Proper Healing^a^	Adolescents, No. (%)		
	All Ages (n = 1293)	Age 10–14 y (n = 836)	Age 15–19 y (n = 457)
Do not have sex	577 (44.6)	242 (28.9)	335 (73.3)
Do not masturbate	319 (24.7)	100 (12.0)	219 (47.9)
Avoid physical activities	252 (19.5)	206 (24.6)	46 (10.1)
Do not touch penis	243 (18.8)	192 (23.0)	51 (11.2)
Change the bandage	224 (17.3)	159 (19.0)	65 (14.2)
Go back to the clinic for checkup	176 (13.6)	121 (14.5)	55 (12.0)
Do not wash genital area with soap	141 (10.9)	107 (12.8)	34 (7.4)
Soak penis in salt water	83 (6.4)	60 (7.2)	23 (5.0)
Practice good hygiene	72 (5.6)	43 (5.1)	29 (6.3)
Do not apply home/folk remedies or agents	67 (5.2)	43 (5.1)	24 (5.3)
Avoid hot temperatures	51 (3.9)	42 (5.0)	9 (2.0)
Follow the instructions	43 (3.3)	29 (3.5)	14 (3.1)
Keep penis facing upright	27 (2.1)	14 (1.7)	13 (2.8)
Take oral medications	27 (2.1)	22 (2.6)	5 (1.1)
Wash the penis with soap	22 (1.7)	10 (1.2)	12 (2.6)
Wear clean pants and underwear	17 (1.3)	9 (1.1)	8 (1.8)
Other	70 (5.4)	50 (6.0)	20 (4.4)
Don’t know	40 (3.1)	37 (4.4)	3 (0.7)

Abbreviation: VMMC, voluntary medical male circumcision.

^a^Open responses to the question “What should a male do right after he is circumcised to ensure proper healing?” Adolescents could provide multiple (unprompted) responses.

When asked to repeat providers’ wound-care instructions during IDIs, both younger and older adolescents gave responses that diverged from guidelines. In South Africa and Zimbabwe, one recommendation is to dip the VMMC wound in salt water solution, prepared with a teaspoonful of coarse salt added to 375 mL of clean water, twice a day for a minimum of 15 minutes. Adolescents, however, mentioned varying amounts of salt and water, as well as means of measurement.

They then said, ‘Before you wash it [wound] with water, you must pour 3 jugs of water and add 3 teaspoonfuls of salt. Thereafter, you dip in the salty water.’ (12- year-old male, Orange Farm, South Africa). Another younger adolescent gave his version. ‘She demonstrated to us that you take an empty jar of peanut butter [375 mL], then you put water and add a tablespoonful of salt and then you dip your organ.’ (13-year-old male, Mutare, Zimbabwe).

In Tanzania, where most participants were reportedly instructed to wash the wound with mild soap, a few claimed that providers had told them to use any type of soap.

I used a soft cloth to clean my wound…. I used a soft cloth and soap…. He [provider] did not specify the type of soap. He said we could just use any soap. (18-year-old male, Iringa, Tanzania).

Dissimilar versions of wound-care instructions suggested that adolescents had either received varied information or had not followed recommended procedures.

Although wound-care instructions were articulated during counseling, younger adolescents (aged 10–14 years) failed to recall these when they got home. In the absence of written materials, some parents/guardians who had not participated in counseling sessions described having to phone or visit VMMC sites to obtain proper wound-care instructions.

He did not do well on the issue of “minutes”…. He would only dip [in salt water solution] for some “seconds.” What then happened is that I went with him on the date of the review that was written on the card. They then explained that it was 15 minutes not 15 seconds…. It was evident he was not healing well. (FGD male parents, Harare, Zimbabwe).

IDIs with adolescents revealed that misrepresenting the instruction about dipping the penis in salt water to parents/guardians was sometimes an intentional attempt to shorten or circumvent the wound-care process. Their accounts suggested that they considered the process frightening, painful, and burdensome.

It [dipping] was quite scary…. At first, I hesitated, so I did so bit by bit and finally dipped the whole of it [penis]…. I had to do it for 15 to 20 minutes, but the first 5 minutes were very painful…. (13-year-old male, Mutare, Zimbabwe).

Because of concerns that their adolescent sons would either fail to recall or intentionally misrepresent wound-care instructions, parents/guardians in all countries expressed a desire to be present during either preoperative or postoperative counseling.

I would like my child to be counseled together with me because he is still young. He could forget some of the things, and I will be able to remind him about what the doctor said if he goes contrary to the doctor’s instructions. (FGD female parents, Iringa, Tanzania).

Older adolescents (aged 15–19 years) believed that the presence of parents/guardians during their counseling session(s) would hinder their ability to freely ask questions, but younger adolescents generally did not mind parents/guardians being present.

### Wound-Care Self-Efficacy and Parental Intervention

Quantitative data suggested that across all countries and age groups, most adolescents felt very confident they could take care of their wound until it completely healed (81.0% of 10–14-year-olds and 89.9% of 15–19-year-olds) (Table 3). However, IDIs suggested that without parental/guardian assistance, both younger and older adolescents were likely to encounter multiple challenges.

**Table 3.** Self-Efficacy for Wound Care Among Adolescent VMMC Clients

Confidence	Adolescents, No. (%)^a^		
	All Ages	Age 10–14 y	Age 15–19 y
**Taking care of wound^b^**	**N = 1287**	**n = 830**	**n = 457**
Not at all confident	28 (2.2)	18 (2.2)	10 (2.2)
Not very confident	49 (3.8)	43 (5.2)	6 (1.3)
Somewhat confident	127 (9.9)	97 (11.7)	30 (6.6)
Very confident	1083 (84.2)	672 (81.0)	411 (89.9)
**Refraining from masturbating/doing self-sex^c^**	**N = 1283**	**n = 826**	**n = 457**
Not at all confident	15 (1.2)	11 (1.3)	4 (0.9)
Not very confident	19 (1.5)	16 (1.9)	3 (0.7)
Somewhat confident	38 (3.0)	23 (2.8)	15 (3.3)
Very confident	478 (37.3)	184 (22.3)	294 (64.3)
*Does not masturbate*	733 (57.1)	592 (71.7)	141 (30.9)
**Refraining from sex for 6 wk after circumcision^d^**	**N = 1288**	**n = 832**	**n = 456**
Not at all confident	7 (0.5)	5 (0.6)	2 (0.4)
Not very confident	16 (1.2)	8 (1.0)	8 (1.8)
Somewhat confident	58 (4.5)	26 (3.1)	32 (7.0)
Very confident	481 (37.3)	157 (18.9)	324 (71.1)
*Not sexually active*	726 (56.4)	636 (76.4)	90 (19.7)
**Refraining from sex for 6 wk even if partner wants to have sex^e^**	**N = 561**	**n = 195**	**n = 366**
Not at all confident	16 (2.8)	10 (5.1)	6 (1.6)
Not very confident	19 (3.4)	7 (3.6)	12 (3.3)
Somewhat confident	56 (10.0)	27 (13.9)	29 (7.9)
Very confident	470 (83.8)	151 (77.4)	319 (87.2)

Abbreviation: VMMC, voluntary medical male circumcision.

^a^Analyses are among all available data for each item.

^b^Survey question: How confident are you that you can manage taking care of your wound for the next 6 weeks until it completely heals?

^c^Survey question: How confident are you that you can refrain from masturbating/doing self-sex for the 6 weeks after your circumcision? (“Although the response item ‘does not masturbate’ was not read by the interviewer, many of the boys stated they did not perform this activity.”)

^d^Survey question: How confident are you that you can refrain from sex for the 6 weeks after your circumcision? (“Although the response item ‘not sexually active’ was not read by the interviewer, many of the adolescents stated they did not perform this activity.”)

^e^Survey question: How confident are you that you can refrain from sex for the 6 weeks after your circumcision even if your partner wants to have sex with you? Interviewers excluded adolescents who reported they were not sexually active in response to the prior question, and another 10–14-year-old participant had missing data for this question.

The one who helped me take care of the wound was my uncle who accompanied me to the facility…. He washed it…. It was helpful because if it was just me washing the wound, I could have done it ineffectively fearing that I would hurt myself. But for him, he did it effectively. (13-year-old male, Njombe, Tanzania).

A younger adolescent described how an older brother’s support had greatly helped him.

He helped me wash the wound at the beginning because I could not wash it properly and blood would clot on it. So he would help me wash it and remove the blood…. The other boys I had gone with told me their wounds were not yet healed when mine was already healed. So I realized that if my brother had not helped me, I was not going to even wash it properly. (12-year-old male, Orange Farm, South Africa).

Older adolescents also acknowledged that parental/guardian intervention helped them overcome potential hindrances to wound care, such as procrastination and/or hesitation.

They would assist me and would ask if I had dipped and so on…. Both my mum and dad…. It helped me because when it is just you, at times you just procrastinate when it is time to dip. Also, you will be like, ‘Eish, it is that time again.’ (16-year-old male, Harare, Zimbabwe).

Parents/guardians sometimes had to solicit external support when an adolescent could not be restrained.

He hesitated to dip in salty water. As a result, his penis did not heal quickly. We ended up being involved as neighbors. We would actually threaten him with a rod. Once he got into the bathroom, we would ensure that he had dipped in salt [salty water]. But he was a 14-year-old boy. (FGD female parents, Mt Darwin, Zimbabwe).

Non–family members were therefore sometimes involved in ensuring compliance with wound-care instructions. Overall, it was suggested that despite initial confidence in self-care, most adolescents required outside assistance to help mitigate challenges and risks associated with wound care.

### Self-Management of Complications and Unsafe Practices

Parents/guardians and adolescents reported behaviors that went against recommendations, such as adolescents exposing their fresh wounds to prove to others they had been circumcised or ignoring symptoms of infection and not returning to the clinic for review when an AE had occurred.

Ah, one will be saying his organ [penis] has grown bigger…. Three of my friends, they will be showing me how different it [penis] has become. We will be showing each other…. One will say, ‘Look at the head [glans] and how it is now.’ (16-year-old male, Mt Darwin, Zimbabwe).

A parent described what she observed:

You would see them showing the others at the bus stop. I don’t know if it was stitches [sutures], because I saw it in passing… It was very scary the way they were showing each other after coming back from the clinic. (FGD female parents, KwaMashu, South Africa).

Despite strong advice to consult a provider in the case of postprocedure complications, adolescents’ accounts insinuated that some had opted to manage these on their own.

When it [wound] formed pus, I drained it…. The next day, I also drained it. That is when it was about to heal, when I removed the stitches…. I removed them on my own. (16-year-old male, Iringa, Tanzania).

Another older adolescent reported avoiding clinical care despite bleeding, swelling, and pain—all of which could be considered either moderate or severe post-VMMC AEs:

The stitches separated and I started bleeding. I went back home and wrapped the wound. My penis then got swollen and I felt pain. I then decided to pull the stitches that I could see…. I was very scared [of going to facility]. I knew they would stitch [suture] me again. (16-year-old male, Mbeya, Tanzania).

For some adolescents, self-management of complications was a strategy to avoid repeating what they believed were painful VMMC procedures.

### Disregarding/Substituting Provider Wound-Care Instructions

Although providers were reported to have emphasized the need to desist from applying substances to wounds, IDIs suggested that some adolescents had disregarded this caution. These adolescents described having applied substances to either ease pain or “disinfect” the wound.

The counselors told me that I was not supposed to wet the wound…. One day as I was urinating, urine passed by the wound and then I put powder [talcum] on it…. Putting powder on the wound honestly helped because I didn’t have any problem with my wound. (19-year-old male, Mbeya, Tanzania).

In other instances, adolescents adopted peer-recommended instructions. An 18-year-old man who had been circumcised using a device reported disregarding the instruction to wash his wound with only soap and adopting what had been recommended to a surgically circumcised peer.

There is this guy who was circumcised here at the hospital, and he was instructed that when he washed, he must wash his penis with salt added to warm water and dip the penis for 15 minutes. I also did that because I was rushing to heal quickly. So I liked to do that when I was going to sleep…. I saw that it worked a lot…. I saw that it worked and it [wound] became cleaner than when you washed with just soap. (18-year-old male, Ermelo, South Africa).

Parents/guardians who had neither attended counseling sessions nor received written wound-care instructions sometimes substituted recommendations. A young adolescent described how his mother had recommended an alternative instruction.

I constantly washed it [wound] with salted water. I told my mother about it. She told me to use sea water instead. (11-year-old male, Umkhanyakude, South Africa).

During FGDs, parents/guardians mentioned that such alternative instructions were often due to past experiences of caring for other wound types (eg, caesarean delivery related).

Instead of putting half a tablespoonful of salt, the mother will say, ‘No! I once had a surgical operation and I would use my palm to measure the salt. You are doing the wrong thing.’ (FGD male parents, Bulawayo, Zimbabwe).

In all countries, FGDs highlighted a lack of knowledge surrounding proper wound care among parents/guardians.

## DISCUSSION

This study reveals important residual gaps in knowledge regarding proper wound-care procedures among adolescent VMMC clients. This was evident through suboptimal knowledge of proper wound care, poor recall of instructions, and reports of incorrect practices. Previously, Kaufman et al reported that younger adolescents had difficulties recalling HIV prevention and health information messages in the context of VMMC [15]. Our findings buttress growing calls to tailor the VMMC service package to the age and information needs of adolescents [1, 5, 15–17]. Innovative and age-appropriate counseling strategies and materials for wound care, especially for younger adolescents, need to be developed and tested.

Given that clients are often reluctant to read instructions [18], one promising approach might be using demonstration videos to help adolescents better comprehend and follow proper wound-care instructions. Furthermore, training for providers in ways to build rapport could alleviate clients’ reluctance to return to the VMMC site for review if complications or AEs requiring clinical management arise. Programs could also consider replacing wound-care requirements considered painful (eg, dipping the penis in a salt water solution) with ones that seem less painful (eg, washing the penis with mild soap). This may be particularly advisable given that fear of pain (including postoperative pain) is a well-recognized barrier to VMMC uptake [14, 19, 20].

Increased parental engagement throughout the adolescent VMMC experience would likely enhance proper wound care [5, 21]. In this study, both adolescents and parents/guardians highlighted several benefits of parental involvement in adolescents’ wound care, including verifying instructions and ensuring adherence. Involving parents/guardians in postoperative counseling sessions, thereby equipping them with knowledge for proper wound-care supervision, would assist them in effectively supporting adolescent adherence to wound-care instructions. However, a key consideration will be how to involve parents/guardians without tempering open discussions around sexuality, especially because older adolescents expressed reservations about parents being present during counseling.

Because non–family members are often involved in wound management, sometimes covertly, interventions to enhance wound care should be designed for both individuals and the community at large. Such interventions would give clear instructions on wound care and genital hygiene, emphasize the benefits of prophylactic tetanus vaccination, and underscore the risks of applying substances and traditional medicines to wounds [9]. Education also needs to emphasize the risks of self-managing complications, which not only may worsen AEs but may also prolong their management and resolution.

This study exploring challenges in postoperative wound care among adolescents has limitations. There may have been selection bias in the survey sample due to loss to follow-up [13, 14]. Because IDIs were conducted 6–10 weeks after VMMC, our findings on counseling about wound care and actual practices during wound care may have been affected by recall bias. However, when poor wound-care practices are reported via IDIs 6–10 weeks after VMMC, they probably reflect suboptimal knowledge of proper wound-care practice 7–10 days after VMMC. In addition, concordance between data obtained through FGDs with parents/guardians and IDIs, along with the similarity of findings across countries, do point to the likely validity and generalizability of the results presented in this study.

Increased involvement of parents/guardians in postprocedure wound-care counseling, including instructions for special supervision of younger adolescents, along with the development of age-appropriate materials on wound care, is needed to minimize postoperative VMMC complications. Preventing AEs is important for maintaining client safety and maximizing the quality, acceptability, and impact of VMMC programs.

## Notes

***Acknowledgments.*** We are grateful to the adolescent participants in this study for sharing their perspectives and experiences. We also thank the VMMC facility managers, community mobilizers, and providers for their support. We acknowledge the contributions of the Technical Advisory Group for the adolescent VMMC assessment and their guidance throughout the study. We are privileged to work with this group of dedicated professionals from PEPFAR, the Centers for Disease Control and Prevention, the US Department of Defense, WHO, the United Nations Children’s Fund, and the Bill and Melinda Gates Foundation. Thank you to the Human Sciences Research Council in South Africa, CSK Research Solutions in Tanzania, PSI/Zimbabwe, and the Centre for Sexual Health & HIV/AIDS Research in Zimbabwe for their assistance with data collection. We appreciate the assistance of Meaghen Murphy with copy editing and Maria Elena Figueroa with project start-up.

***Financial support.*** This work was supported by USAID with PEPFAR funding (Cooperative Agreement AID-OAA-A-12-00058) to the Johns Hopkins Center for Communication Programs and cofunded by the UK Department of International Development through the Integrated Support Program in Zimbabwe.

***Supplement sponsorship.*** This article appears as part of the supplement “Adolescent Voluntary Medical Male Circumcision: Vital Intervention Yet Improvements Needed,” sponsored by Johns Hopkins University.

***Potential conflicts of interest.*** All authors: No reported conflicts of interest. All authors have submitted the ICMJE Form for Disclosure of Potential Conflicts of Interest. Conflicts that the editors consider relevant to the content of the manuscript have been disclosed.
